# Delayed Presentation of Jejunal Atresia

**DOI:** 10.34763/devperiodmed.20172102.9597

**Published:** 2017-08-11

**Authors:** Charu Sharma, Hemanshi Shah, Mukta Waghmare, Jayesh Desale, Pankaj Dwivedi

**Affiliations:** 1 Dept of Paediatric Surgery, TNMC & BYL Nair Hospital, Mumbai Central, Mumbai, Maharashtra. India. Pin: 400008

**Keywords:** obstruction, sub-acute, jejunal web, central perforation

## Abstract

Type I intestinal atresias (webs) are rare causes of gastrointestinal obstruction in infants, the most common site being the second portion of the duodenum. According to the Louw and Barnard classification, type 1 atresia has been defined as an intra-luminal web which results in either complete (web with no perforation) or incomplete (web with central perforation) intestinal obstruction. The jejunum is a rare site of such webs. Diagnosis of an incompletely obstructing web due to central perforation is usually difficult and challenging. We present two cases of jejunal web with a central perforation in which the presentation was delayed. Both were managed by excision of the web.

## Introduction

Type I intestinal atresias (webs) are rare causes of gastrointestinal (GI) obstruction in infants [1]. The most common site is the second portion of the duodenum where it is often associated with trisomy 21, cardiac or renal anomalies [1]. The jejunum is a rare site of such webs. A large study from Taiwan has reported only 8% of the webs arising from the jejunum [1, 2]. The presenting symptoms of the fenestrated mucosal web are sub-acute intestinal obstruction with poor weight gain in children [1]. Diagnosis is difficult [1, 2]. We present two cases of jejunal web with central perforation and delayed presentation.

## Case summary

Informed consent was taken from the parents of both the patients.

### Case 1

**Fig. 1:**
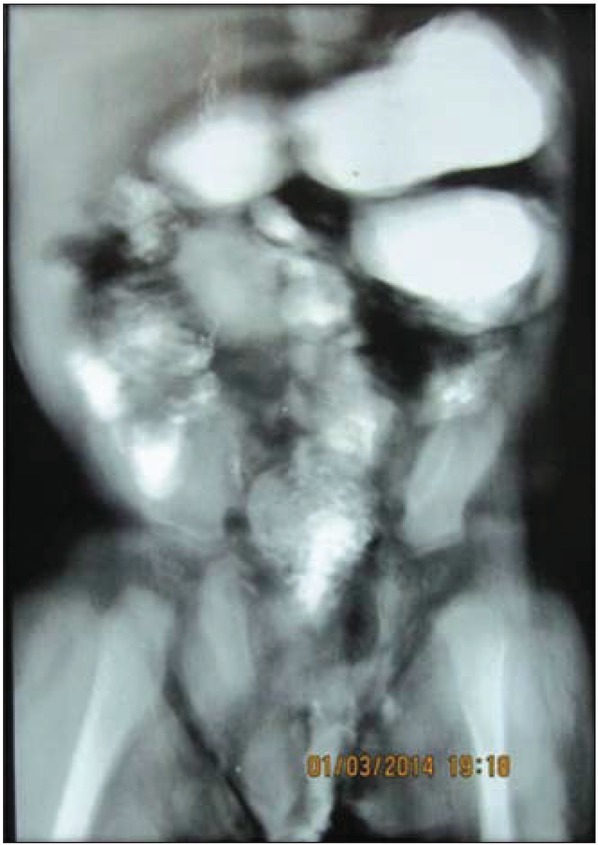
Delayed plate of an upper gastrointestinal contrast study showing a distended stomach and persistently dilated duodenum.

**Fig. 2:**
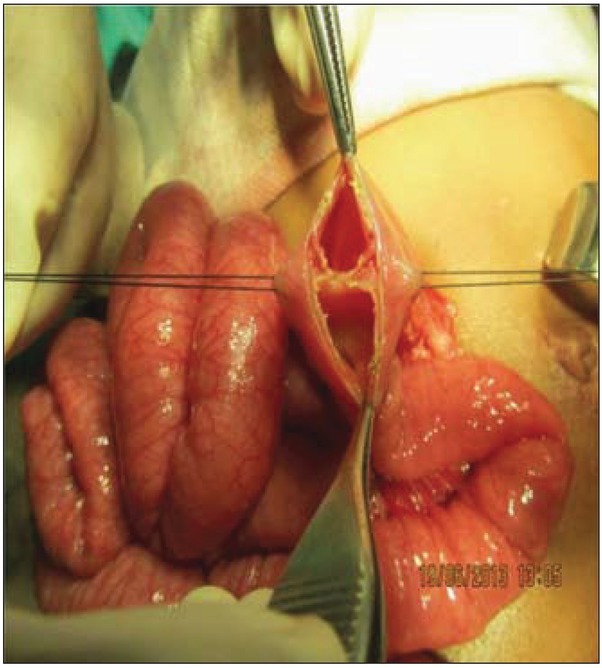
Intra-operative image of the first patient showing the jejunal web.

An 8-month-old male child presented with intermittent episodes of bilious vomiting after starting a semisolid diet at 5 months. The abdomen was soft with no distention or visible peristalsis. Plain erect x-ray findings were unremarkable. An upper gastrointestinal contrast study revealed a distended stomach and duodenum with persistent dilatation of the duodenum in delayed plates (Fig. 1). Abdominal ultrasound (USG) and Contrast Enhanced Computed Tomography (CECT) revealed a distended stomach and duodenum with no evidence of malrotation.

after taking informed consent, the patient was taken up for surgery. At diagnostic laparoscopy, the stomach and proximal jejunum were distended. At laparotomy, a jejunal web located 12 cm from the duodeno-jejunal junction was found (Fig. 2). The web was excised and distal patency was confirmed. Histopathology was consistent with jejunal mucosa.

### Case 2

A 2-year-old emaciated male child presented with intermittent episodes of bilious vomiting of one year’s duration. He had also presented with failure to thrive and poor weight gain.

**Fig. 3:**
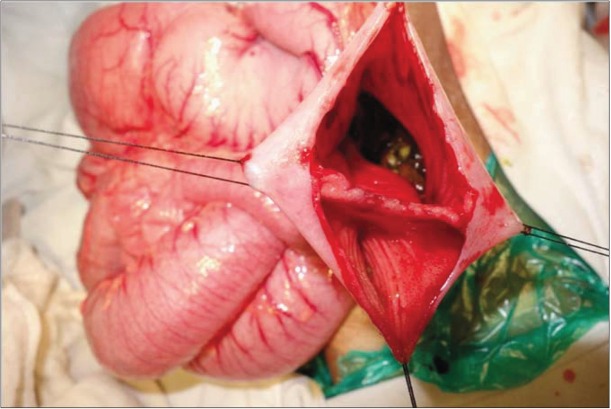
Intra-operative image of the second patient showing the jejunal web.

A plain erect x-ray of the abdomen showed a grossly dilated stomach and upper duodenum. An upper gastrointestinal contrast study revealed a distended stomach and duodenum with persistent dilatation of the duodenum in delayed plates. Abdominal USG and CECT revealed a dilated first and second part of the duodenum. There was no evidence of malrotation. after taking informed consent, the patient was taken up for surgery. At laparotomy, a jejunal web with central perforation was found approximately 15 cm from the duodeno-jejunal flexure (Fig. 3). The web was excised.

## Discussion

Jejuno-ileal atresias have been classified as type I (mucosal web), type II (atretic fibrous cord), type IIIa (V-shaped mesenteric defect), type IIIb (apple peel atresia), and type IV (multiple atresias) [3]. Type 1 atresia, according to the Louw and Barnard classification [1, 4] has been defined as an intra-luminal web which results in either complete (web with no perforation) or incomplete (web with central perforation) intestinal obstruction [1]. The jejunum is a rare site of such a web. In a jejunal web with central perforation, the clinical presentation is variable and the diagnosis often difficult and delayed [5].

The exact etiology of intestinal atresias is unknown. Louw and Barnard proposed vascular accidents as a cause, especially ones associated with gastroschisis [4, 6]. However, this theory fails to explain vascular events in otherwise normal children [6].

Other factors like vasoconstricting medications, toxins and cystic fibrosis have also been related to jejuno-ileal atresias [6]. Recently, it has been suggested that disruption in endodermal development results in intestinal atresias [6]. Mutations in the endodermal Fgfr2IIIb gene or its encoding ligand Fgf10 have been found to result in both colonic and duodenal atresias [6]. Disruption of Hedgehog signaling proteins produced in the fetal endoderm or mutations of a gene encoding Cdx-2, an endodermal transcription factor, may also result in intestinal atresia [6].

The extent of clinical symptoms is proportionate to the diameter of the perforation in the web [5]. Presentation is usually delayed with features of intermittent vomiting, failure to thrive, poor weight gain and emaciation [1, 5, 6]. A plain erect X-ray shows a hugely dilated stomach and proximal bowel. Contrast studies reveal a persistently dilated stomach and proximal bowel with delayed transit of the contrast in delayed films.

Review of the literature suggests few reports and studies on jejunal atresias. Andrews and Stem, De Backer et al, Kothari et al, Seltz and Baba et al [5] have reported jejunal webs in their studies which were managed by laparotomy and excision [5]. Rudolph et al [6] have reported jejunal web to be associated with intestinal hyperproliferation [6]. Recently, Tang [1] has reported a jejunal web causing diagnostic confusion as intestinal malrotation in a neonate [1]. The clinical presentation of sub-acute obstruction coupled with radiology often falls short of the correct diagnosis in atresias with central perforation.

The management of jejunal web is surgical excision, even though variable approaches may be used [5]. Endoscopic laser therapy, as well as simultaneous laparotomy with endoscopy has also been reported [5, 7].

Though rare, jejunal web with perforation should be considered as a differential in patients presenting with longstanding sub-acute intestinal obstruction in childhood.
